# Measles outbreak response immunization during the COVID-19 pandemic: lessons from Borno State, Nigeria

**DOI:** 10.11604/pamj.2022.41.104.28162

**Published:** 2022-02-06

**Authors:** Terna Nomhwange, Abede Mohammed, Anne Eudes Jean Baptiste, Audu Musa, Abdulhakeem Yusuf, Mohammed Yusuf, John Wagai, Aliyu Shettima, Sule Meleh, Richard Banda, Fiona Braka, Richard Luce, Balcha Masresha

**Affiliations:** 1 World Health Organization, Country Office, Abuja, Nigeria,; 2 World Health Organization, Borno State Office, Maiduguri, Nigeria,; 3 State Primary Health Care Development Agency, Borno State, Nigeria,; 4 World Health Organization, Inter-country Support Team for West Africa, Ouagadougou, Burkina Faso,; 5 World Health Organization, African Regional Office, Brazzaville, Congo

**Keywords:** Measles, outbreak response, immunization, Borno, COVID-19, Nigeria

## Abstract

**Introduction:**

the COVID-19 outbreak was declared a public health emergency of international concern by the WHO on the 30^th^ January 2020. The occurrence of measles outbreaks in the context of COVID-19, both highly infectious respiratory illnesses, impacts additional challenges to the health system in a state with an ongoing humanitarian crisis. This article documents the implementation of an outbreak response immunization (ORI) during the COVID-19 pandemic and the implementation of global guidelines for mass vaccination.

**Methods:**

a retrospective review of the response to measles outbreak implemented in Borno state across six local government areas (LGAs) in 2019 was conducted. This review assessed the utilization of the World Health Organization (WHO) decision making framework, measles and COVID-19 epidemiological reports and the measle's vaccination response data.

**Results:**

an outbreak response immunization was implemented in six LGAs in Borno State, with a validated post campaign coverage of 96.3% (95% CI: 93.0 - 98.1). In total, 181,634 children aged 9 months-9 years were vaccinated with 27,961 (15.4%) receiving the measles vaccine for the first time. Prior to the interventions, 20 COVID-19 cases were reported in the six LGAs while only seven suspected cases were reported with only two cases confirmed in one of the six LGAs four weeks after the ORI.

**Conclusion:**

the WHO decision-making framework for implementing mass vaccinations in the context of the COVID-19 Pandemic was utilized for the outbreak response immunization in Borno State, Nigeria with 181,634 children aged 9 Months-9 years vaccinated with the measles vaccine. The use of the WHO decision-making framework to assess risk benefits of initiating mass vaccination campaigns remains a very important practical tool. These types of responses in Nigeria and other low and middle income countries (LMICs), with hitherto suboptimal immunization coverage and weak health systems and other settings, affected by humanitarian emergencies is essential in the achievement of the regional measle's elimination targets.

## Introduction

New and emerging diseases continue to occur globally with significant impact on global health security. The novel Coronavirus (COVID-19) outbreak was declared a public health emergency of international concern (PHEIC) by the World Health Organization (WHO) on January 30^th^ 2020, and has continued to spread worldwide. This has put a strain on public health systems and economies across the world. The similarity of the current outbreak and the Spanish flu pandemic in 1918 continues to be discussed [1,2]. Nigeria reported its first COVID-19 case on the 27^th^ of February 2020. The outbreak has since then, spread to over 34 states (out of the 36 states) plus the federal capital territory (FCT) as of May 2020. Over 15,000 COVID-19 cases were confirmed as at June 8^th^, 2020, with 367 of these in Borno State. More than a year after, over 200,000 cases have been reported by the Nigeria centre for disease control (NCDC). The Borno State COVID-19 index case was reported on 18^th^ of April 18^th^, 2020 (epidemiological week 16). As of October 3^rd^, 2021, 1352 of these were reported in Borno state. The COVID-19 outbreak in Borno is occurring in the context of an already challenging humanitarian emergency [3]. The COVID-19 pandemic has had multiple effects on the continuity of the immunization programs especially in low and middle-income countries and increase the risks for vaccine-preventable diseases (VPDs) outbreaks due to disruption in the demand and delivery of immunization services [4].

Nigeria has experienced repeated outbreaks of measles in recent years primarily due to low routine immunization (RI) coverage. In 2019, Borno State reported over 15,000 suspected cases and 75 measles deaths across 22 local government areas [5,6]. In the initial 15 epidemiological weeks of 2020, a total of 1,198 suspected measles cases and 7 measles related deaths had been reported across 20 LGAs, with outbreaks confirmed in 18 communities/wards of six LGAs. The occurrence of measles outbreaks in the context of COVID-19, both highly infectious respiratory illnesses, possess an additional challenge to the fragile health system in a state already impacted by a humanitarian crisis resulting from an ongoing insurgency over the past nine years [7-9]. Multiple global and regional guidelines have been developed to support the continuous provision of services in the context of the COVID-19 pandemic, and reduce the risk of transmission of the disease among health workers and clients presenting to health facilities seeking care [10,11]. The guidelines regarding preventive mass vaccinations and outbreak response activities pivot around the risk benefit analysis of implementing mass vaccination interventions in the context of COVID-19. Modelling studies have shown that the benefit of implementing routine immunization services outweighs the risk of COVID-19 infection in an immunization clinic setting, especially in African countries [12]. This article seeks to document the implementation of a measles outbreak response immunization (ORI) in Borno State, Nigeria following the use of the WHO decision-making framework within the context of the COVID-19 pandemic, and the implementation challenges vis-a-vis the global and regional guidelines for immunization.

## Methods

**Study setting:** Borno is one of six North eastern states in Nigeria, covering an area of 57,799 square kilometres (Km^2^) with an estimated population of 11.5 million (Polio immunization plus days - IPDs extrapolated). It shares international borders with Cameroun, Chad, and Niger Republic; and within the country, with Adamawa, Gombe and Yobe States. Borno consists of 27 local government areas (LGAs) of which four were reported as totally inaccessible; five having all their wards fully accessible; and the remaining 18 partially accessible (with some wards, settlements or communities either partially or fully inaccessible).

**Decision-making process:** the WHO decision-making framework for the conduct of outbreak response campaigns in the context of COVID-19 was used to assess the overall benefit of an ORI to reduce measles mortality in an emergency situation compared with the potential risk of increased COVID-19 transmission [10]. The stepwise decision making process.

**Population immunity and measles risk:** a measles risk analysis was conducted based on the national measles risk assessment template as guided by WHO, in January 2020 [13]. The measles risk assessment was conducted focusing on 21 variables from 4 thematic areas; population immunity, surveillance quality, programme delivery performance and threat probability assessment.

**Measles and COVID-19 epidemiological situation:** an LGA was declared to have a confirmed measles outbreak if within the same month it reported 3 or more Immunoglobulin (IgM) positive measles cases, as per the Regional measles surveillance guidelines [14]. Laboratory confirmation was done using measles IgM testing of serum specimens collected within the first 30 days after the onset of rash in suspected cases. A confirmed case of measles by epidemiological linkage was defined as a suspected case of measles that has not been confirmed by the laboratory but was geographically and temporally related to a laboratory-confirmed case or another epidemiologically linked measles case, with dates of rash onset occurring 7-23 days apart. A suspected case with clinical symptoms of measles, but no adequate specimen taken and not linked epidemiologically to a laboratory-confirmed case was classified as a clinically compatible case [14]. We reviewed the measles case-based surveillance data, the outbreak investigation report and the line-list data to characterize the outbreak. We also analysed the epidemiological characteristics and trends of measles by epidemiological week and conducted a root-cause analysis to identify factors which may have led to the outbreak. The number of COVID-19 positive cases were extracted from Nigeria´s centre for disease control (CDC) COVID-19 daily situation reports and from the state laboratory results. Nasopharyngeal and oropharyngeal samples were collected and transported, in reverse cold chain within a viral transport media to a designated COVID-19 testing Labouratory. samples were collected only from suspected persons that met the standard case definition for COVID-19.

**Administrative data tools:** designated health facilities were supported to set up additional temporary outreach posts to reach the eligible population within their catchment area, while ensuring strict maintenance of social distancing, minimal waiting time, and zero tolerance for overcrowding. The administrative data was collected for all age group targeted (9 months-9 years) on the provided RI intensification data tools and disaggregated by age group, sex and previous vaccination status. Additional information was documented on the regular health facilities routine immunization (RI) data tools for children within the RI age group (9-11 months). The number of children vaccinated as part of the outbreak response immunization efforts were documented using tally sheets, while the number of vaccine doses provided were documented at the point of service delivery and compiled at the LGA and state level. All data was collated and analyzed using microsoft® Excel spreadsheet.

**Vaccination survey:** ward micro-plans which include the details of the number of settlements in a ward and the estimated settlement population were used as the sampling frame for this survey. Settlements were selected as the primary sampling unit. In the selected settlements, all households were line-listed and the line list was used to select households for interviewing. Multistaged sample selection method was used. In the first stage, 20 settlements were randomly selected from each of the six LGAs out of a total of 923 settlements. In the second stage 7 households were randomly selected in each settlement, providing a total of 840 households in the six LGAs. The third stage involved the random selection of one child for interviewing from each household. Android based mobile phones were used to collect data using ODK-based (Open data kit) application. Data collection was done over a period of five (5) days. Range checks and skip patterns were coded into the data entry program to ensure that only all valid responses were collected and that there were responses to all applicable questions. On completion of the household roaster, only one age-eligible respondent (between 9 months-9 years) was presented for interviewing in a household. Once an interview was completed, data from an enumerator´s phone was synchronized with a centralized server. Data cleaning and analysis was conducted using the supplementary immunisation activity (SIA) module of vaccination coverage quality indicators (VCQI) software running on Stata version 15 (Stata Corp. 2017. Stata Statistical Software: Release 15. College Station, TX: Stata Corp LLC).

## Results

**Figure 1:**
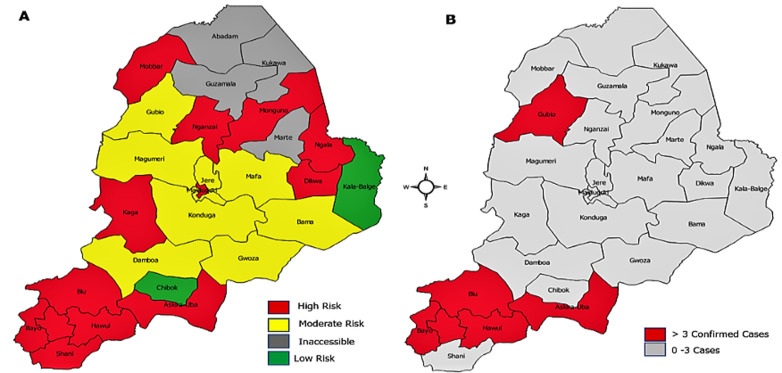
measles risk status, January 2020 A) and local government areas with measles outbreak (> 3 Laboratory-confirmed cases/month) as at April 2020 B) - Borno State, Nigeria 2020

**Population immunity and measles risk:** the risk assessment classification for each LGA is indicated in Figure 1. In January 2020, the state was considered to be at high risk for measles: 12 LGAs and the Maiduguri metropolitan city (MMC) were found to be at high risk and 8 were categorized as medium risk. Two LGAs were categorized at low risk for measles. Five out the 12-high risk LGAs and a medium risk LGAs (Gubio) reported confirmed measles outbreaks by April 2020 (Figure 1).

**Figure 2:**
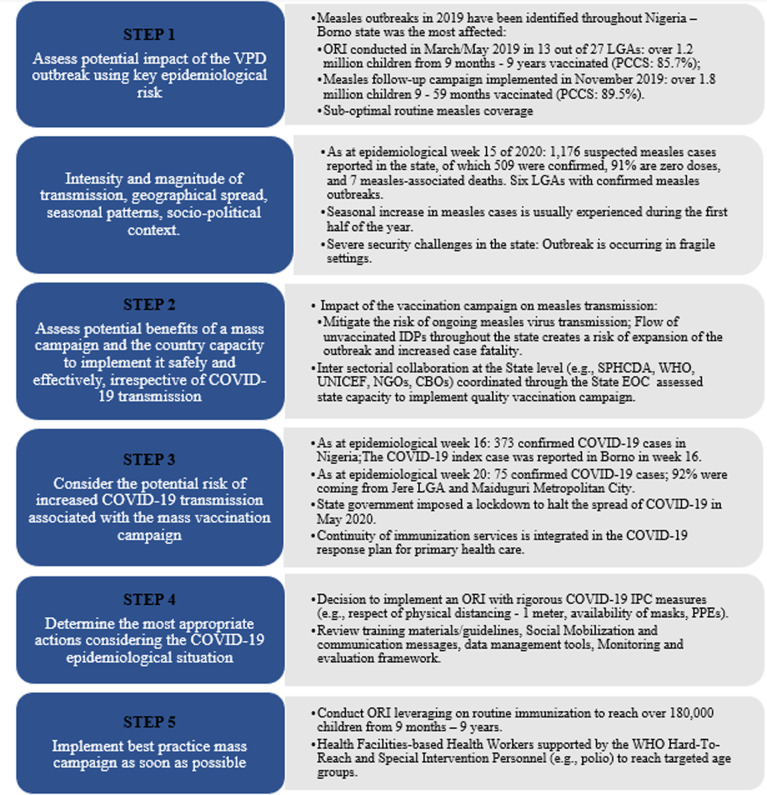
measles outbreak response immunization during COVID-19 decision-making flowchart-Borno State, Nigeria 2020

**Decision-making process:** the World Health Organization, following a review of the effect of the COVID-19 pandemic on immunization and VPD control efforts, recommends the utilization of a decision-making framework to assess risk benefits of initiating mass vaccination campaigns. The tool allows for implementing officers to make objective decisions to reduce risk of spread of the SARS-CoV-2 if immunization campaigns are to be implemented. The review of the epidemiological risk for VPD outbreak was considered as “very high” while the COVID-19 transmission scenarios was considered as “sporadic cases”. Following the framework, the state followed the five steps as outlined in Figure 2. The Borno team following steps 1 and 2 concluded that a mass vaccination campaign was beneficial to controlling the measles outbreak and that a high-quality vaccination could be implemented with adequate IPC measures. The state team also reviewed the COVID-19 epidemiology in the state and possible options for implementing a vaccination campaign. Considering the risk of the measles outbreak and spread within an already vulnerable population within a humanitarian crisis, the conclusion was to conduct an outbreak response immunization (Figure 2).

**Figure 3:**
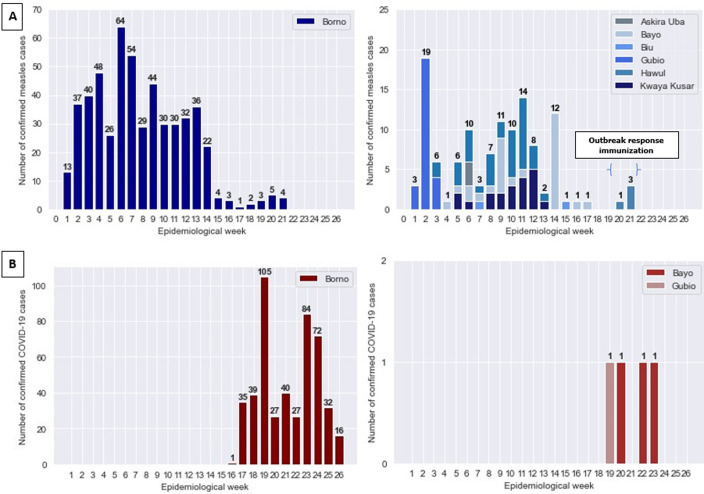
distribution of measles; A) and COVID-19 cases; B) as at epidemiological week 26-Borno State, Nigeria 2020

**Measles and COVID-19 epidemiological situation:** the epidemiological situation of measles and COVID-19 by LGAs are shown on Figure 3A. As at epidemiological week 15 of 2020, Borno State had reported a total of 1,176 suspected measles cases, of which 509 were confirmed measles cases (56 laboratory-confirmed, 50 epidemiologically linked and 403 clinically compatible with measles) with 7 measles-associated deaths. Six (6) LGAs (Askira uba, Bayo, Biu, Gubio, Hawul and Kwaya Kusar) met the criteria for confirmed measles outbreak (Figure 1). The investigation report of the confirmed cases showed the age group of the suspected cases to be within the range of 9-108 months. It further indicated that 91% had never been vaccinated with measles vaccine (excluding the unknown vaccination status). The root-cause analysis revealed that children that had not received the measles vaccine doses were mostly from nomadic population with a few falling into the non-compliant/vaccine hesitant cases recorded in a recent measles follow-up campaign conducted in November 2019.

**Outbreak response planning:** the plan for measles outbreak response in Borno State endorsed by the State Primary Health Care Development Agency (SPHCDA) was based on WHO recommendations of risk-benefit assessments and included interventions for the protection of health workers (HW) and populations during the planned interventions [11]. The vaccination strategies adopted in the outbreak response plan included the following. i) Vaccination campaign in affected LGAs using the health facilities HWs as well as Hard-to-Reach (HTR) and special intervention personnel engaged by WHO on an ad hoc basis, to reach the targeted age group over an extended period of 10-14 days. ii) Additional temporary outreach sites and sessions for the affected settlements/communities for RI. iii) Pre-positioning of Measles-containing-vaccine (MCV) with the special intervention teams at transit points and along known nomadic routes in all identified HR LGAs. iv) Integration of vaccination activities with other essential health services including antenatal care services and malnutrition assessment.

The ORI targeted 181,465 children from 9 months-9 years. Response activities were conducted in the 18 wards of the six LGAs with confirmed measle's outbreak. The targeted population for all the LGAs used for the measles outbreak response immunization (ORI) was estimated using a combination of data from the Polio IPDs (the highest ever number of children immunized), geographical information systems (GIS) settlement imagery as part of the microplanning process outputs and IOM Reports. These were the only reliable sources of data in the state due to the dynamic nature of the population. A population of 181,465children aged 9 months-9 years was used for estimation of number of vaccination post, vaccines and other logistics requirements in all the six LGAs. Daily implementation plans were developed by all teams to cover all settlements through fixed, and outreach strategies. Trainings for all health workers, recorders and other team members were conducted at the LGA level. These trainings focused on the administration of measles vaccines, use of tally sheets for data collection and infection prevention control (IPC) measures. Health workers were updated about the COVID-19 situation and how to prevent the spread of the disease using infection prevention control measures and effective communication with caregivers during the vaccination activities. The State deployed 134 vaccination teams (82 Fixed/Health Facilities and 52 Temporary Outreach Posts) for the ORI activities within a period of ten implementation days. All team personnel were provided with face masks, gloves and hand sanitizers. Social distancing (a minimum physical distance of 1 meter between clients) was emphasized at the service delivery points. The deployed teams comprised of 2 HWs, 2 Recorders, 1 Social Distance enforcer, and 1 Mobilizer. All health posts were provided with personal protective equipment (PPEs) supplies, including face masks and alcohol-based hand sanitizers. The vaccinators did not wear gloves, unless the skin of the children had a lesion, rash, or cut. Caregivers were also sensitized on the preventive measures against COVID-19 infection. The ORI was fully supervised by the state and LGA teams as well as supporting partner agencies to ensure smooth conduct and strict adherence to COVID-19 prevention principles.

**Outbreak response immunization:** the implementation took place between 13^th^ - 22^nd^ May 2020. The duration of the planned ORI ranged between 6 - 11 days across the implementing wards. Two wards (Teli and Briyel) in Bayo LGA conducted a mop-up exercise on day 11 to reach the targeted coverage of 95%. All the health facilities in the implementing wards were optimized during the ORI. The facility provided RI services irrespective of their initial schedule. A functional health facility was available in 17 out of the 18 affected wards. In one partially accessible ward with no functional health facility, vaccination activities were conducted with the support of civilian joint task forces (CJTFs) and only Temporary Outreach Posts were deployed.

**Advocacy, communication and social mobilization:** community engagement and communication strategies were carried-out with the community heads within the affected settlements. Clear messaging to affected communities was propagated on the reason for the response.

**Figure 4:**
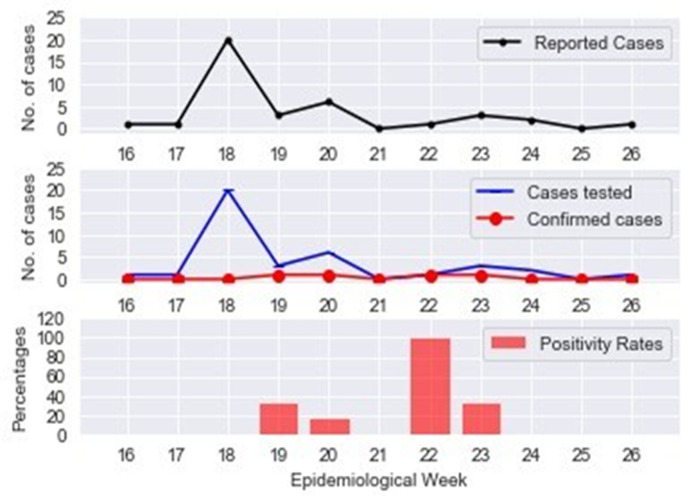
weekly distribution of COVID-19 cases, tests conducted and positivity rates in the six LGAs with measles outbreaks - epidemiological week 16-26

**Outcome of response interventions:** a total of 181,634 children (administrative coverage: 100.1%) were vaccinated of which 27,961 (15.4%) received the measles vaccines for the first time. The administrative coverage varied across LGAs, with the lowest coverage (98.0%) observed in Gubio LGA (Table 1). All the eighteen wards that were involved in the ORI were considered for the post campaign coverage survey. However, the reporting domain was at the LGA level (one administrative level higher than the ward). Five out of six LGAs involved in the ORI achieved measles vaccination coverage of 95% or more. The aggregated weighted coverage for the eighteen wards included in the survey was 96.3% (95% CI: 93.0 - 98.1). The distribution of COVID-19 cases in Figure 3B demonstrate that at the time of the ORI activities (2020 epidemiological week 20), Borno State had reported 207 COVID-19 cases across 11 LGAs; Bayo, Biu, Gubio, Gwoza, Jere, Konduga and MMC, the State capital and largest city. Two COVID-19 cases had already been confirmed from 2 of the LGAs (Bayo and Gubio) where the response activities were initiated. The proportion of confirmed COVID-19 cases out of those tested is as shown in Figure 4. The period with highest number of COVID-19 cases reported in the six LGAs where the ORI was implemented was in epidemiological week 18 with 20 cases reported and tested. In the weeks after the implementation (epidemiological week 22 - 26), only seven suspected cases were reported with two COVID-19 cases confirmed in one LGA (Bayo) after the ORI.

**Table 1:** age category of children immunized with measles vaccine during the outbreak response immunization, Borno State, 2020

LGA	Targe population (9 months - 9 years)	Total vaccinated (9 months - 9 years)	% Admin coverage	Zero dose children reached (9 months - 9 years)	% Zero dose reached out of total vaccinated
Askira/Uba	46,226	47,956	103.7%	1,959	4.1%
Bayo	43,035	42,463	98.7%	6,163	14.5%
Biu	45,588	45,231	99.2%	15,215	33.6%
Gubio	5,081	4,977	98.0%	732	14.7%
Hawul	24,739	24,333	98.4%	363	1.5%
Kwaya Kusar	16,797	16,674	99.3%	3,529	21.2%
Borno	181,466	181,634	100.1%	27,961	15.4%

LGA; local government area

## Discussion

The implementation of a high-quality mass vaccination response to measles and other VPDs is possible within the context of the COVID-19 pandemic in Nigeria and other LMICs. We have documented that 181,364 children were vaccinated in Borno state in May 2020, with 15% of these children vaccinated for the first time (Zero Dose children). We also have shown that the measles risk assessment remains a useful tool for program implementation, as the areas highlighted as high risk for measles transmission align with the areas where measles outbreaks were reported in Borno state as at week 15 of 2020. The quality of the campaign, validated at 96.3% coverage by survey, shows the capacity to conduct high-quality outbreak responses in areas of ongoing humanitarian crises as currently occurring in NorthEast Nigeria as well as the ongoing COVID-19 pandemic. The impact of this response is seen in the Borno State measles epidemiology reports, with a decline in measles cases in the states and in the six affected LGAs after the ORI implementation in Epi-weeks 20-21 of 2020. While the concern for mass vaccinations as a risk for further spread of COVID-19 is genuine, the utilization of the WHO decision-making framework for the implementation of mass vaccinations and the adaption of operationalization measures to reduce risk of exposure to HWs and communities can go a long way in limiting VPD outbreaks, while at the same time reducing the spread of COVID-19. The available epidemiology of SARS-CoV-2 disease in Borno state shows no increase in cases after the response activity. The implementation of all response activities must be guided by recommended infection prevention and control (IPC) guidelines to ensure health workers remain comfortable and assured of their safety to be able to deliver vaccines in a safe and controlled manner. The provision of additional requirements for IPC and Personal protective equipmen (PPEs) for the response results in additional costs and must be considered as countries implement various campaigns in the context of COVID-19. This additional costs have been estimated to result in as much as a 49% increase in operational cost in low intensity scenarios [15].

The use of the WHO decision-making framework to assess risk benefits of initiating mass vaccination campaigns remains a very important tool. This tool allows for implementing officers in the field to make objective decisions to reduce risk of spread of the SARS-CoV-2 disease if immunization campaigns are to be implemented. The successful outcome of this activity has an impact on encouraging the continuation of other delayed vaccination activities and mass campaigns, not only for measles but for other vaccine-preventable diseases (VPDs). The decision to implement outbreak response and other vaccination activities is based on the benefit-risk of intervening to a VPD related death and that from SARS-CoV-2 disease. While this may be a difficult task in some countries without available evidence, a modeling study by Abbas *et al*. (2020) already shows that the direct and indirect benefits to vaccinations (all vaccines and measles vaccine specifically) outweigh risk of SARS-CoV-2 deaths with a benefit-risk ratio of 96 for Nigeria and an MCV1 benefit-risk of 103 [12]. The opportunities of response activities allow for increased engagements and risk communication around issues of COVID-19 infection to high-risk populations in humanitarian crises, as recommended by the United Nations Office for the coordination of humanitarian affairs (OCHA) [16]. Challenges of vaccination in areas with security concerns leveraged on lessons learned from the GPEI program to ensure all children in these areas were vaccinated. This includes the support of the military and civilian joint task force (CJTFs) to move with teams to ensure access in partially accessible areas. The integration of immunization with other services provided in the humanitarian settings such as treatment points, food delivery points, financial payment points etc. are very important in addressing refusals/hesitancy and increasing vaccine uptake.

**Limitations:** our study did not evaluate the impact of the response on improved awareness of the COVID-19 based on the information shared by HWs during the campaign. We may have missed an opportunity to do this during the conduct of the post-implementation vaccination survey and recommend the inclusion of survey questions to better understand community concerns of implementing vaccinations during the COVID-19 pandemic and its effect on vaccine acceptance or refusals. The study is limited by the generalization of the findings with the sporadic case transmission scenario of COVID-19 in the LGAs where the response activity was implemented and may not be applicable to high transmission/community transmission settings. However, this emphasizes the need for the utilization of the decision-making framework for the implementation of any vaccination activity and the postponement of any activity if could have a negative effect. Although there was no increase in the number of COVID-19 cases in the implementing LGAs and communities almost 4 weeks after the response, the subsequent increase in cases later in the year in Borno State, may need further detailed epidemiologic description of cases. The global guidelines for mass vaccination in the context of COVID-19 pandemic and the decision-making framework are useful tools, and when used properly, can result in the conduct of vaccination activities with little risk to health workers and transmission of the Corona virus to communities. In many of these countries, the decision to intervene is based on a benefit-risk assessment of VPD outbreaks control and SARS-CoV-2 disease. While some studies available show the direct benefit of vaccination outweighs the risk, this is even more so for measles vaccination which accounts for a huge number of under 5 years´ deaths in Nigeria and Africa

## Conclusion

The WHO decision-making framework for implementation of mass vaccinations in the context of the COVID-19 Pandemic was utilized for the outbreak response immunization in Borno State, Nigeria provided an opportunity for a supplementary measle's vaccination dose. The use of the WHO decision-making framework to assess risk benefits of initiating mass vaccination campaigns remains a very important practical tool. Implementation of ORI activities are critical to containing controlling VPDs outbreaks, including measles, resulting from the increasing number of susceptible persons due to interruption of immunization services and broader essential primary health care activities due to the COVID-19 pandemic. The importance of this response in Nigeria and other LMICs, with hitherto suboptimal immunization coverage and weak health systems, and other settings affected by humanitarian emergencies is important in the achievement of the regional measle's elimination targets.

### What is known about this topic

- Measles is a vaccine preventable disease with a tendency to spready especially in settings with low immunization coverage below 95% expected to achieve herd immunity;
- COVID-19 pandemic has impacted negatively on essential health services including immunization;
- Implementation of immunization response to outbreaks in humanitarian settings remains important in controlling spread of the infection and reduction of morbidity and mortality especially in children under five years in Africa.

### What this study adds

- Documentation of the practical use of the WHO COVID-19 decision making framework in addressing a measles outbreak and implementation of a vaccination response;
- Field lessons from the outbreak response of measles in a level 3 humanitarian crisis during the covid 19 pandemic in Borno state, Nigeria;
- Implementation of vaccination response during the COVID-19 pandemic in Nigeria and the efforts towards strengthening adherence of use of PPEs and IPCs to reduce risk of exposure to communities and infection of health care workers (HCW).
